# Host response to immune checkpoint inhibitors contributes to tumor aggressiveness

**DOI:** 10.1136/jitc-2020-001996

**Published:** 2021-03-11

**Authors:** Irina Khononov, Eyal Jacob, Ella Fremder, Nili Dahan, Michal Harel, Ziv Raviv, Boris Krastev, Yuval Shaked

**Affiliations:** 1 Rappaport Faculty of Medicine, Rappaport Technion Integrated Cancer Center, Technion Israel Institute of Technology, Haifa, Israel; 2 OncoHost LTD, Binyamina, Israel; 3 Clinic of Medical Oncology, MHAT Hospital for Women Health Nadezhda, Sofia, Bulgaria

**Keywords:** immunotherapy, immunomodulation, drug therapy, combination, cytokines

## Abstract

**Background:**

Immune checkpoint inhibitors (ICIs) have made a paradigm shift in clinical oncology due to unprecedented long-term remissions. However, only a small proportion of patients respond to ICI therapy. It is, therefore, essential to understand the mechanisms driving therapy resistance and to develop strategies for increasing response rates. We previously demonstrated that in response to various cancer treatment modalities, the host activates a range of biological processes that promote tumor regrowth and metastasis. Here, we characterize the host-mediated response to ICI therapy, and investigate its contribution to therapy resistance.

**Methods:**

Tumor cell migration, invasion and motility were assessed in the presence of plasma from ICI-treated mice and patients. Immune cell composition in peripheral blood and tumors of ICI-treated mice was assessed by flow and mass cytometry. Plasma host factors driving tumor aggressiveness were identified by proteomic profiling, followed by bioinformatic analysis. The therapeutic effect of inhibiting host-mediated processes in ICI-treated mice was assessed in a tumor model.

**Results:**

Tumor cells exhibit enhanced migratory and invasive properties in vitro on exposure to plasma from anti-PD1-treated mice. Moreover, mice intravenously injected with plasma-exposed tumor cells display increased metastatic burden and mortality rate in comparison to control arms. Furthermore, tumors from anti-PD1-treated mice as well as Matrigel plugs containing plasma from anti-PD1-treated mice are highly infiltrated with immune cell types associated with both antitumor and protumor activity. These collective findings suggest that anti-PD1 treatment induces a systemic host response that potentially counteracts the drug’s therapeutic activity. Proteomic profiling of plasma from anti-PD1-treated mice reveals an activation of multiple biological pathways associated with tumor aggressiveness. Consequently, blocking IL-6, one of the key drivers of the identified biological pathways, counteracts ICI-induced metastatic properties in vitro and improves ICI treatment efficacy in vivo. Lastly, plasma samples from ICI-treated non-small cell lung cancer patients differentially affect tumor cell aggressiveness in vitro, with enhanced tumor cell motility correlating with a worse clinical outcome.

**Conclusions:**

ICI therapy induces host-mediated processes that contribute to therapy resistance. Identification and analysis of such processes may lead to the discovery of biomarkers for clinical response and strategies for overcoming therapy resistance.

## Background

The discoveries of immune checkpoint molecules have led to the development of a new class of cancer immunotherapies in the form of immune checkpoint inhibitors (ICIs).^1^ These agents have revolutionized cancer treatment as the focus of treatment has shifted from the tumor itself to the host’s immune system. The first immune checkpoint proteins that were discovered include cytotoxic T-lymphocyte-associated protein 4 (CTLA-4), programmed cell death protein-1 (PD-1) and its ligand, PD-L1. These proteins, which are expressed by immune cells (CTLA-4, PD-1) and tumor cells (PD-L1), play key roles in promoting cytotoxic T lymphocyte (CTL) exhaustion, inhibiting T cell-mediated cytotoxicity and allowing tumor cell immune evasion.^2^ Therapeutic antibodies targeting these immune checkpoint proteins (ie, ICI therapy) have shown promising and remarkable successes for the treatment of advanced malignancies such as melanoma, non-small cell lung cancer (NSCLC), renal cell carcinoma and some hematological cancers.^2–6^ However, therapeutic benefit is limited to only a small proportion of treated patients, with the majority considered to be resistant to such therapies.^7^ In addition, several common cancer types such as breast, prostate and colon cancers have shown very low frequency of response to ICI therapy.^8^ Thus, biomarkers of both resistance and response to ICI therapies are critically needed for guiding clinical decisions and optimizing treatment plans for individual patients.

It has been suggested that PD-L1 expression, mutational burden, and mismatch repair deficiency in tumors represent predictive biomarkers for clinical outcome of ICI therapy.^8–13^ Other explored biomarkers are related to tumor-infiltrating immune cells such as T cells (in their different phenotypic states),^14^ immunosuppressive macrophages and myeloid derived suppressor cells (MDSCs).^15 16^ However, despite intensive efforts in this direction, biomarkers available today for guiding clinical decisions are suboptimal in terms of their predictive value.^17^

Our previous studies have demonstrated that in response to various types of anticancer treatment modalities, including chemotherapy,^18^ radiation,^19^ surgery^20^ and molecularly targeted drugs,^21^ the host generates protumorigenic biological processes, which can promote tumor regrowth and metastasis.^22^ In this study, we ask whether resistance to ICI therapy may be explained, in part, by host-mediated effects that occur in response to therapy, similar to the cases reported for other anticancer treatment modalities.^23^ Using tumor cell lines and experimental tumor models in mice, we show that the host response to ICI therapy involves a release of host-derived factors into the circulation which directly contribute to tumor aggressiveness. Our findings suggest that analyzing such host responses in a clinical setting may be relevant for the clinical management of ICI-treated cancer patients.

## Methods

### Tumor cell cultures

EMT6 breast carcinoma, B16 melanoma and Lewis lung carcinoma (LLC) lung carcinoma from murine origin as well as A549 human NSCLC cell lines were purchased from the American Type Culture Collection (Manassas, VA) and were used within 6 months of resuscitation. RET murine melanoma cells were obtained from Prof. Neta Erez (Tel Aviv University, Israel). The cells were cultured in Dulbecco’s modified eagle medium supplemented with 10% fetal bovine serum), 1% L-glutamine, 1% sodium-pyruvate and 1% penicillin–streptomycin (Biological Industries, Israel). Cells were cultured at 37°C in 5% CO_2_ and were tested to be mycoplasma-free.

### Drugs

Anti-PD1 (10 mg/kg, RMP1-14, BioXcell, Lebanon, New Hampshire, USA), anti-CTLA-4 (10 mg/kg, 9H10, BioXcell) or IgG isotype control (10 mg/kg BioXcell) were injected intraperitoneally every other day to BALB/c or C57BL/6 mice, as indicated in the text. In some experiments, anti-interleukin 6 (IL-6) (10 mg/kg MP5-20F3, BioXCell) was administered every other day.

### Animal models

Mice were purchased from Envigo Israel, and were housed in SPF conditions. For primary tumor growth studies, EMT6 (0.5×10^6^) cells were injected into the mammary fat pad of 8-week-old BALB/c mice. Tumor size was assessed regularly with Vernier caliper using the formula width^2^ ×length×0.5. On day 7 postimplantation, treatment with IgG (control), anti-CTLA-4, anti-IL-6 or a combination of anti-CTLA-4 and anti-IL-6 antibodies was initiated. Survival was monitored. Mice were sacrificed when tumor size reached 1500 mm^3^.

For experimental lung metastasis studies, EMT6 cells tagged with luciferase, RET, B16 or Lewis lung carcinoma (LLC) cells were cultured for 4 hours in the presence of plasma from mice that had been treated with IgG (control), anti-PD1, anti-interleukin-6 (IL-6) or a combination of anti-PD-1 and anti-IL-6 antibodies, as indicated in the text. Cells were washed extensively and then injected into the tail vein of BALB/c or C57BL/6 mice (5×10^4^ cells/mouse) as follows: EMT6 cells were injected to BALB/c mice whereas B16, RET and LLC were injected to C57BL/6 mice. Mice-bearing EMT6 tumors were analyzed by in vivo imaging system (IVIS) for metastatic burden in the lungs, and bioluminescence measurements were calculated. Mouse survival was monitored over time in all tumor models.

For plasma collection, tumor-free or tumor-bearing BALB/c or C57BL/6 mice, as well as tumor-free severe combined immunodeficiency (SCID) mice, as indicated in the text, were treated with IgG, anti-PD1 anti-CTLA-4 or anti-IL-6 antibodies. In the case of tumor-bearing mice, tumor cell lines, as indicated in the text, were subcutaneously implanted (5×10^5^ cells/mouse), and when tumors reached 500 mm^3^, treatment with the relevant antibodies was initiated. One week later, mice were sacrificed, blood was drawn by cardiac puncture into EDTA tubes and plasma was separated. Plasma was stored at −80°C until use.

### Blood collection from NSCLC patients

The human study was approved by the ethics committee at MHAT Hospital for Women Health Nadezhda, Sofia, Bulgaria, after patients signed an informed consent. NSCLC patients who were enrolled to the study were undergoing anti-PD1-based or anti-PD-L1-based immunotherapy (n=10). Patient characteristics are defined in online supplemental table S1. Blood samples were obtained by standard blood draws into EDTA tubes at baseline (ie, before commencement of treatment) and on-treatment (ie, 2–3 weeks after the first therapy dose, when the patient visited the clinic for the next therapy dose). Plasma was separated, and stored at −80°C until use.

10.1136/jitc-2020-001996.supp1 Supplementary data

### In vitro invasion migration and scratch wound assays

The effect of plasma on invasion and migration using the Boyden chamber assay, and tumor cell motility using the scratch wound assay was carried out as previously described.^18 24^ Detailed information is provided in online supplemental materials.

### Matrigel plug assay

Matrigel (0.5 mL) was mixed with plasma obtained from IgG- or anti-PD1-treated tumor-bearing mice (10:1, Matrigel:plasma, by volume). The mixture was subcutaneously injected into the flanks of BALB/c or C57BL/6 female mice (n=3–5 mice/group). Plugs were removed 10 days later, and were subsequently prepared as single cell suspensions for flow cytometric analysis (described below) or processed for histological analysis as follows. Matrigel plugs were embedded in 10% paraformaldehyde at room temperature for 24 hours. Next, plugs were embedded in O.C.T (Tissue-Tek) at 4°C for 48 hours and frozen at −80°C. The frozen plugs were sectioned (10 µm thick) using a cryostat, and stained with H&E. Images were captured using a Leica CTR 6000 microscope in bright field.

### Flow cytometry and mass cytometry analyses

The assessment of different immune cells in Matrigel plugs, peripheral blood and tumors was carried out using flow cytometry and mass cytometry as previously described.^25^ Flow cytometry was performed to validate the mass cytometry results. Detailed information is provided in online supplemental materials.

### Cell viability by AlamarBlue assay

Cell viability was assessed using the metabolic indicator dye AlamarBlue (AbD Serotech, Oxon Kidlington, UK) as described.^18^ Detailed information is provided in online supplemental materials.

### Protein array and ELISA

Plasma proteins of tumor-free BALB/c mice treated with IgG, anti-PD1 or anti-CTLA-4 antibodies was assessed by protein arrays and/or specific ELISAs. Detailed information is provided in online supplemental materials.

### Statistical analysis

To ensure adequate statistical power, all experiments were performed with at least two technical repeats and three biological repeats. In the in vitro studies, analysis was performed on at least three biological repeats and >4 fields/group were assessed. In the in vivo experiments, number of mice per group were indicated in the figure. In addition, the mice that exhibited pathological conditions unrelated to the experiment were excluded from the analyses. All experiments were performed in a randomized manner. Data are presented as mean ± SD. The in vivo experiments were repeated twice (n>5 mice/group). Statistically significant differences were assessed by one-way analysis of variance, followed by Tukey post hoc test (when comparing between more than two groups) using GraphPad Prism V.4 software (La Jolla, California, USA). When applicable, estimate of variance was performed and statistical significance comparing only two sets of data was determined by two-tailed Student’s t-test. Significance was set at ps<0.05, and designated as follows: *p<0.05; **p<0.01; ***p<0.001.

## Results

### A host-mediated response to ICIs promotes tumor cell aggressiveness

**Figure 1:**
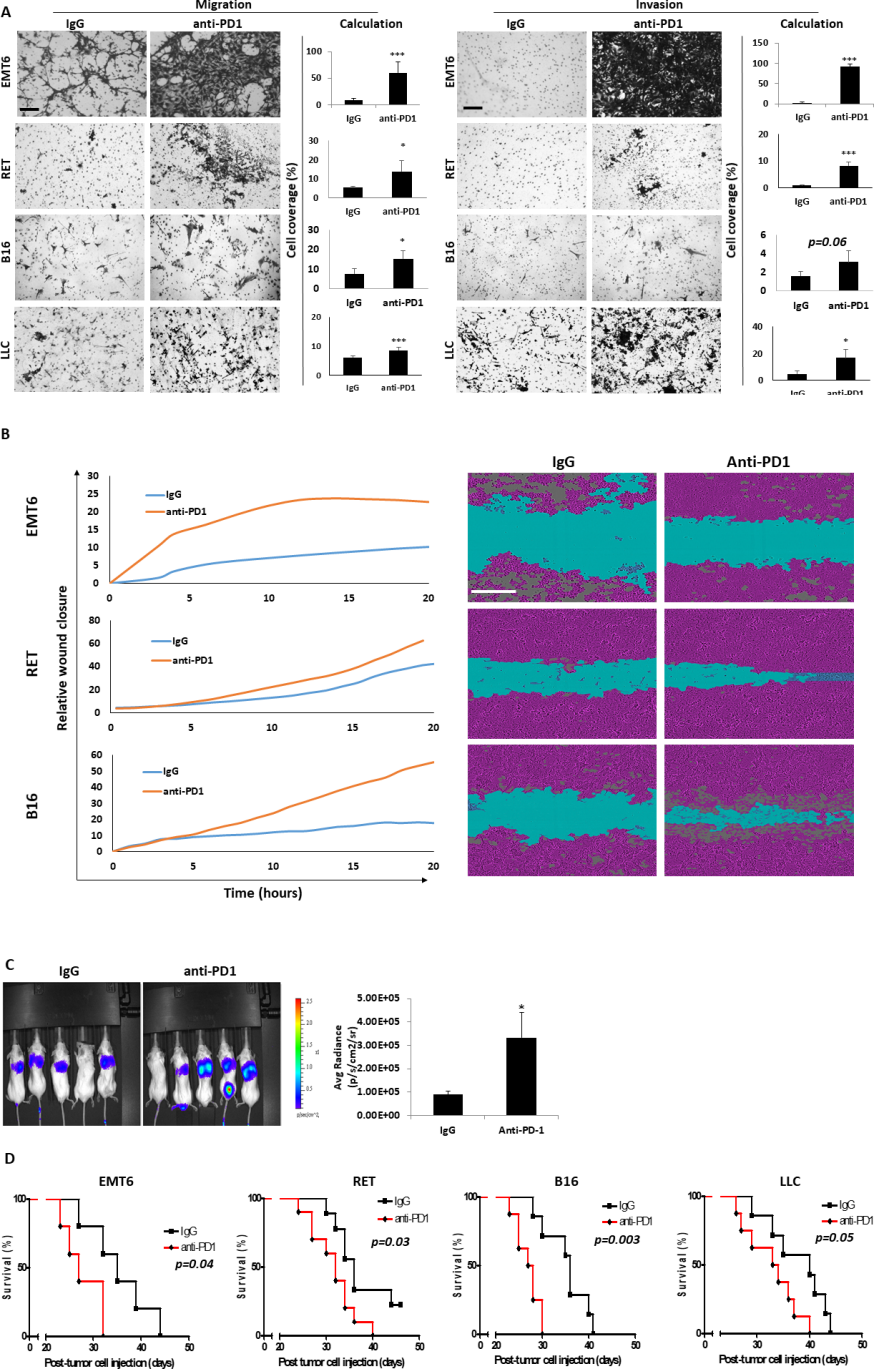
Plasma from anti-PD1-treated mice enhances the metastatic properties of tumor cells. EMT6, RET, B16 and LLC tumor-bearing mice were treated with anti-PD1 or control IgG antibodies. One week later, mice were sacrificed, and plasma was collected. (A) Migratory (left) and invasive (right) properties of EMT6, RET, B16 and LLC cells were assessed in Boyden chamber assays in the presence of plasma. Representative images are shown. Scale bar, 200 µm. Cell coverage was quantified from the images (n=5–8 fields/ group). (B) Motility properties of EMT6, RET and B16 cells were assessed by a scratch wound assay in the presence of plasma extracted from IgG-treated or anti-PD1-treated tumor-free mice. Gap closure was monitored over time by IncuCyte imaging (left). Images at the 20-hour time point are shown (right). Scale bar 300 µm. (C) EMT6 cells tagged with luciferase were cultured for 4 hours in the presence of plasma extracted from IgG-treated or anti-PD1-treated tumor-free mice. the cells were then injected through the tail vein of naïve mice to form pulmonary metastasis. Three weeks later, mice were imaged by IVIS (left) and bioluminescence was quantified (right). (D) EMT6, RET, B16 and LLC cells were cultured for 4 hours in the presence of plasma extracted from IgG-treated or anti-PD1-treated tumor-free mice. The cells were then injected through the tail vein of naïve mice, and survival was monitored. Kaplan-Meier curves are shown. Statistical significance was assessed by unpaired two-tailed t-test. Significant p values are shown as *p<0.05; **p<0.01. ***p<0.001. IVIS, in vivo imaging system.

Previous studies have identified therapy-induced, host-mediated mechanisms that may explain resistance to various cancer treatment modalities.^22 23^ To characterize the host-mediated response to ICI therapy, we first asked whether anti-PD1 treatment induces a systemic host response that promotes tumor cell aggressiveness. To this end, mice were implanted with EMT6, RET, B16 or LLC cells, and when tumors reached a size of 500 mm^3^ treatment with anti-PD1 or IgG control antibodies was initiated. After 1 week, plasma was collected and used in a variety of assays. Plasma from anti-PD1-treated mice increased the migratory and invasive properties of all tested cell lines in vitro, in comparison to plasma from control IgG-treated mice (figure 1A). Similar effects were observed with plasma from anti-CTLA-4-treated mice, suggesting that the effect is not limited to PD-1/PD-L1 axis inhibition (online supplemental figure S1A). Of note, adding anti-PD1 or IgG antibodies directly to EMT6 cultures or as chemotaxis molecules did not affect migration and invasion of the cells, ruling out the possibilities that the effects observed in the presence of plasma are simply due to a direct effect of the antibodies or chemotaxis (online supplemental figure S1B).

We next asked whether the enhanced migratory and invasive activities in the presence of plasma are due to tumor-derived or host-derived prometastatic factors released into the circulation following anti-PD1 treatment. To distinguish between these two possibilities, similar experiments to those described above were performed using plasma from tumor-free mice. Migration and invasion of EMT6 cells were enhanced in the presence of plasma derived from tumor-free, anti-PD1-treated mice in comparison to controls (online supplemental figure S1C), similar to the effect of plasma from tumor-bearing, anti-PD1-treated mice. In addition, plasma from tumor-free, anti-PD1-treated mice enhanced cell motility of EMT6, RET and B16 cells, as demonstrated by faster wound closure in a scratch wound assay (figure 1B), suggesting that host-secreted factors, rather than tumor-secreted factors, are mostly responsible for the tumor cell aggressive effects. Collectively, these experiments demonstrate that systemic host-derived factors induced by ICI treatment promote tumor cell aggressiveness in vitro.

Next, to evaluate whether this systemic host-mediated response affects tumor aggressiveness in vivo, EMT6 luciferase-tagged cells were precultured with plasma from tumor-free mice treated with anti-PD1 or IgG control antibodies and subsequently injected through the tail vein to naïve mice. As demonstrated by IVIS imaging, lung metastasis was significantly increased in the anti-PD1 group in comparison to control (figure 1C). In agreement with these findings, mice injected with EMT6, RET, B16 or LLC cells that were pre-cultured with plasma from anti-PD1-treated mice exhibited an increased mortality rate in comparison to the respective control groups (figure 1D). In a different experiment, mice were directly treated with anti-PD1 or IgG control antibodies, and 1 week later, intravenously injected with tumor cells to generate pulmonary metastasis. In this case, mortality rate was similar in the two groups for all cell lines tested (online supplemental figure S2). It is possible that, in this experimental setup, host-induced tumor aggressiveness is counteracted by the therapeutic effect of anti-PD1 pretreatment in vivo. Overall, our findings from in vitro and in vivo experiments suggest that anti-PD1 treatment induces a systemic host-mediated response involving circulating factors that in turn promote tumor cell aggressiveness.

### Anti-PD1 treatment increases the mobilization and homing of cells associated with protumor and antitumor immunity

**Figure 2:**
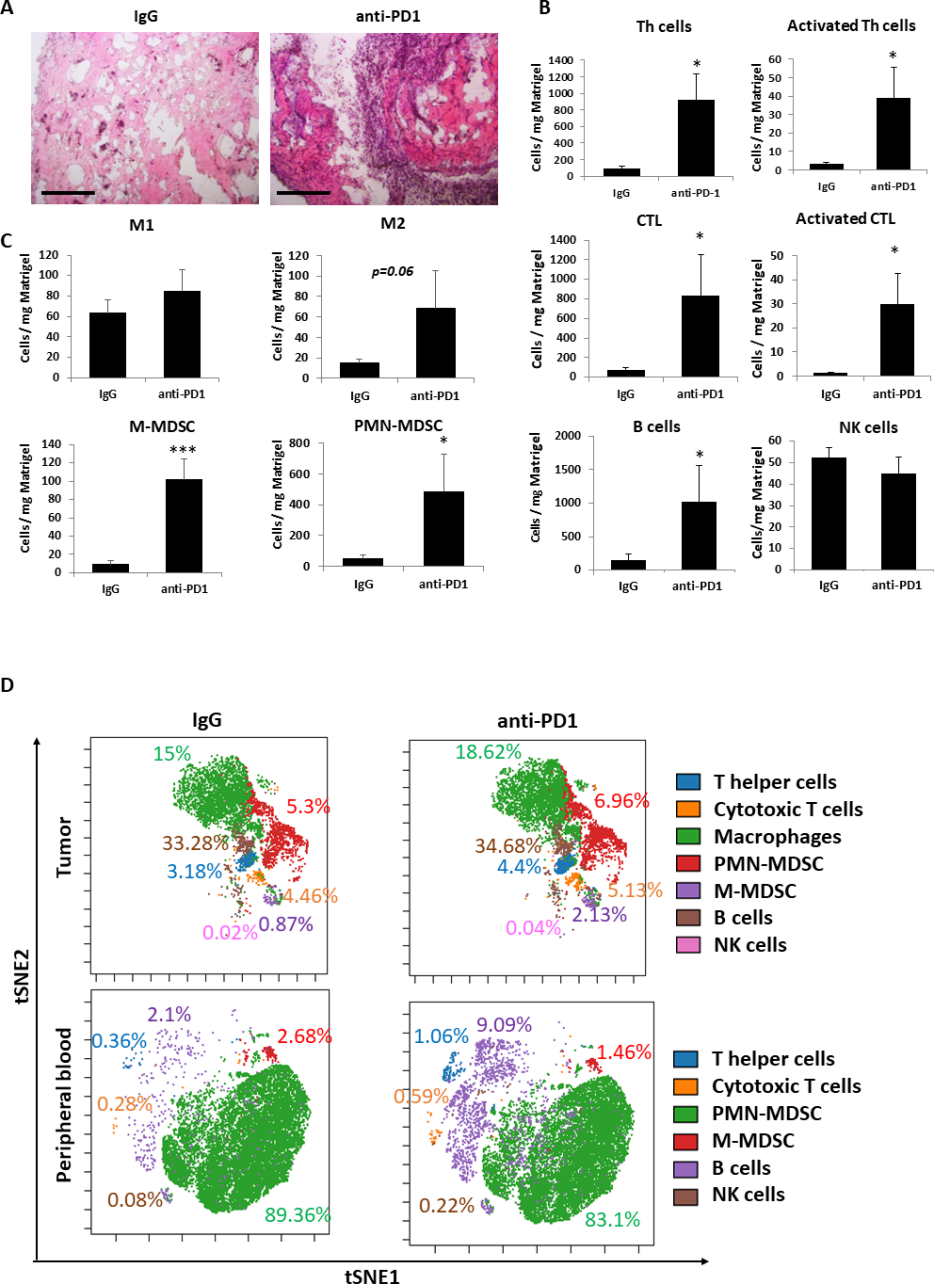
Anti-PD1 treatment induces the recruitment of protumor and antitumor immune cells. (A–C) EMT6 tumor-bearing BALB/c mice were treated with IgG control or anti-PD1 antibodies. One week later, mice were sacrificed, and plasma was collected. The plasma was mixed with matrigel in a 1:10 ratio and the mixture was implanted into the flanks of naïve BALB/c mice. After 10 days, matrigel plugs were removed, sectioned and stained with H&E. Representative images are shown. Scale bar 200 µm (A). In a parallel experiment, matrigel plugs were prepared as single cell suspensions and evaluated by flow cytometry. Absolute numbers of lymphoid (B) and myeloid (C) immune cells per Mg matrigel are presented. (D) EMT6 tumor-bearing BALB/c mice were treated with IgG control or anti-PD1 antibodies. One week later, blood was drawn, and tumors were removed and prepared as single cell suspensions. Immune cell composition of peripheral blood samples (pooled per group) and tumor cell suspensions (pooled per group) were anaylzed by cytometry by time of flight (CyTOF). Percentages of each immune cell type out of total immune cells are shown. CyTOF data were validated by flow cytometry (shown in online supplemental figure S4). Statistical significance was assessed by unpaired two-tailed t-test. Significant p values are shown as *p<0.05; ***p<0.001. CTL, cytotoxic T lymphocyte; PMN-MDSC, polymorphonuclear myeloid derived suppressor cell; M-MDSC, monocytic myeloid derived suppressor cell; M1, M1-like macrophage; M2, M2-like macrophage; NK, natural killer.

We and others have previously demonstrated that the systemic host response to various cancer treatments is accompanied by acute mobilization of bone marrow derived cells (BMDCs) from the bone marrow compartment and their homing to the treated tumor site where they support protumorigenic activities.^26 27^ For example, following chemotherapy, BMDCs home to the treated tumor site and support angiogenesis and epithelial to mesenchymal transition.^18 28^ Similarly, angiogenesis-supporting BMDCs were found to home to Matrigel plugs containing plasma from chemotherapy-treated mice.^18^ We, therefore, sought to identify the immune cell types that are associated with the host response to anti-PD1 treatment. To this end, plasma extracted from anti-PD1- or IgG-treated tumor-bearing mice was mixed with Matrigel and implanted in naïve mice. After 10 days, the plugs were removed and analyzed. H&E staining revealed increased infiltration of host cells in plugs containing plasma from anti-PD1-treated mice (figure 2A and online supplemental figure S3A). Increased numbers of active Th cells and CTLs were observed in plugs of the anti-PD1 group, as demonstrated by flow cytometry analysis (figure 2B and online supplemental figure S3B). However, concurrently, this group also exhibited increased numbers of immunosuppressive M2-like macrophages and both monocytic and granulocytic MDSCs (figure 2C and online supplemental figure S3C), all of which are known to inhibit cytotoxic immune cell activity.^29^

To gain further insight into the effect of anti-PD1 treatment on immune cell composition in tumor bearing mice, peripheral blood and tumors extracted from anti-PD1-and IgG-treated EMT6 tumor bearing mice were analyzed by cytometry by time of flight (CyTOF) followed by flow cytometry validation. Comparable to the Matrigel plug assay, an increase in the number and activity of some of the immune cell populations including Th cells and CTLs were found in peripheral blood and tumors of mice treated with anti-PD1 compared with control mice (figure 2D and online supplemental figure S4). In addition, the numbers of immunosuppressive cells such as M2 macrophages and monocytic MDSCs were increased in tumors of anti-PD1-treated mice (online supplemental figure S4). These collective findings suggest that both antitumor and protumor immune activities occur on anti-PD1 treatment.

### The protumorigenic response to ICI therapy is primarily mediated by cells of the adaptive immune system

**Figure 3:**
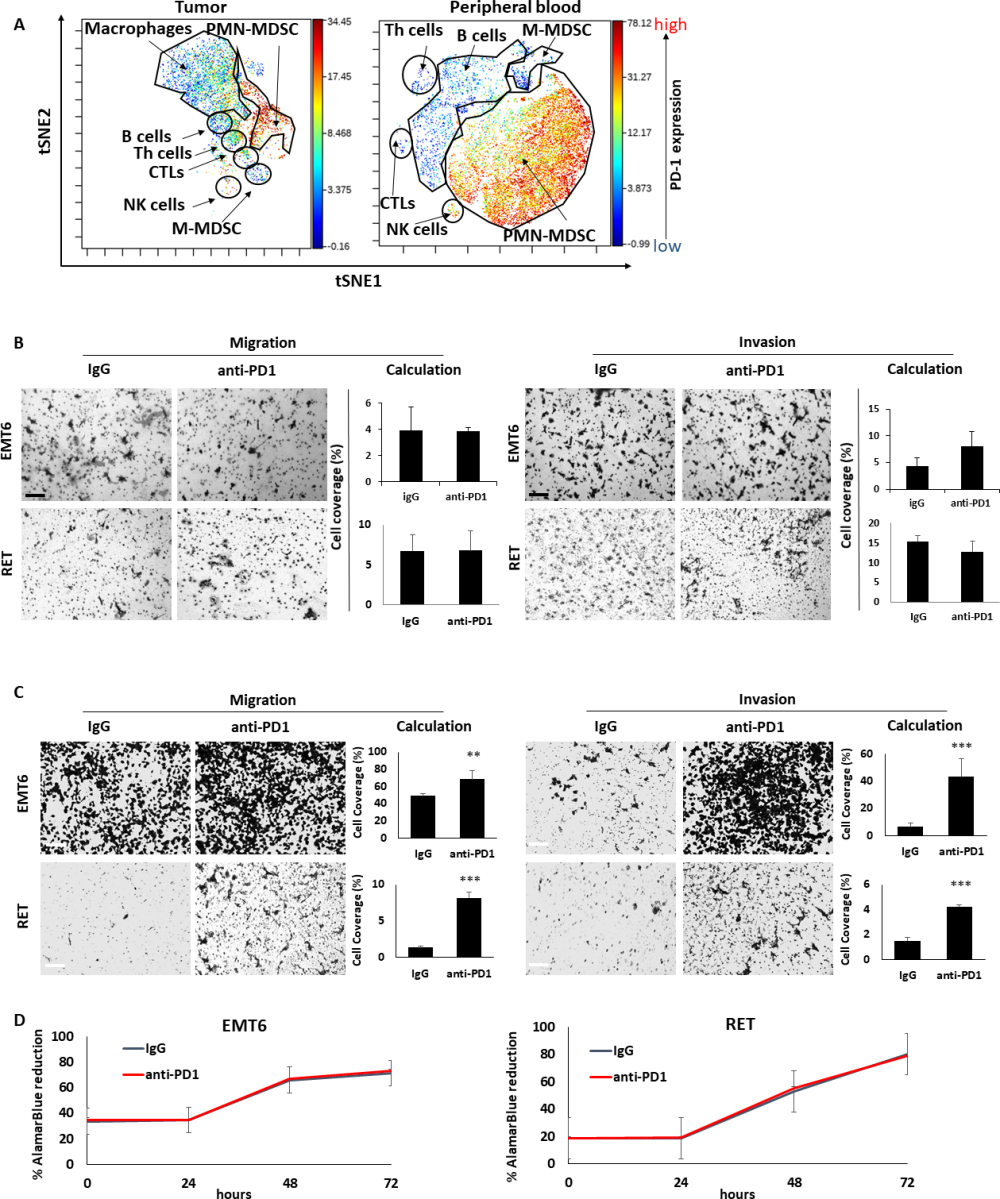
The prometastatic host response to anti-PD1 therapy is dependent on the adaptive immune system. (A) EMT6 tumors and peripheral blood were obtained from BALB/c mice and single cell suspensions were prepared. The samples were pooled and subsequently acquired by CyTOF to evaluate the expression level of PD1 in the different immune cell subsets. (B) Tumor-free SCID mice were treated with anti-PD1 or control IgG antibodies. One week later, mice were sacrificed, and plasma was collected. Migratory (left) and invasive (right) properties of EMT6 and RET cells were assessed in Boyden chamber assays in the presence of plasma extracted from IgG-treated or anti-PD1-treated mice. Representative images are shown. Scale bar, 200 µm. Cell coverage was quantified from the images (n=5–8 fields/group). (C, D) CD8 T cells were isolated from the spleens of anti-PD1 or IgG-treated EMT6-bearing BALB/c mice and RET-bearing C57BL/6 mice. Conditioned medium (CM) was collected from T cell cultures. The effect of the CM on migration (left) and invasion (right) of EMT6 or RET cells was assessed by Boyden chamber assays. Representative images are shown. Scale bar, 200 µm. Cell coverage was quantified from the images (n=5–8 fields/ group) (C). EMT6 or RET cells were cultured in the presence of CM. Cell viability was assessed by AlamarBlue assay (D). Statistical significance was assessed by unpaired two-tailed t-test. Significant p values are shown as **p<0.01; ***p<0.001. CTL, cytotoxic T lymphocyte; Th, T helper; PMN-MDSC, polymorphnuclear myeloid derived suppressor cell; M-MDSC, monocytic myeloid derived suppressor cell; NK, natural killer.

Thus far, our findings suggest that anti-PD1 treatment induces a systemic host-mediated response that occurs independently of the tumor, ultimately promoting tumor aggressiveness. We, therefore, hypothesized that the response is initiated by PD1-expressing host cells. Indeed, recent studies have demonstrated that PD1 is expressed by various immune cells including T cells and a subset of myeloid cells.^30^ Based on CyTOF data, we found that PD1 is expressed not only by CTLs, but also by B cells, macrophages as well as granulocytes in the blood but not in the tumor (figure 3A), in line with previous publications.^30–32^ Thus, it is possible that anti-PD1 treatment affects PD1-expressing ‘on-target’ CTLs or other ‘off-target’ immune cell types, in a manner that ultimately contributes to tumor aggressiveness.

To assess the contribution of CTLs to the protumor host response, SCID mice, which lack an adaptive immune system,^33^ were treated with anti-PD1 or IgG control antibodies, and plasma was extracted 1 week later. Focusing on breast cancer and melanoma models, the effect of the SCID mice plasma samples on the migratory and invasive properties of EMT6 and RET cells was assessed in vitro. No differences in cell migration and invasion were observed when comparing the effects of plasma from anti-PD1- and IgG-treated SCID mice (figure 3B). This contrasts with the results from the same experiment performed in immunocompetent mice (figure 1A), suggesting that the adaptive immune system is necessary for tumor-promoting, host-mediated effects induced by anti-PD1 treatment. In agreement, conditioned medium of CD8+ T cells obtained from spleens of anti-PD1-treated immunocompetent mice enhanced migratory and invasive properties of EMT6 and RET cells in comparison to conditioned medium of T cells from IgG-treated control mice (figure 3C). Of note, possible direct effects of the antibodies on cell viability were ruled out (figure 3D). Taken together, these findings suggest that CD8 +T cells play a major role in mediating protumorigenic activities in response to anti-PD1 treatment.

### Plasma proteomic profiles following anti-PD1 therapy are associated with protumorigenic biological pathways

**Figure 4:**
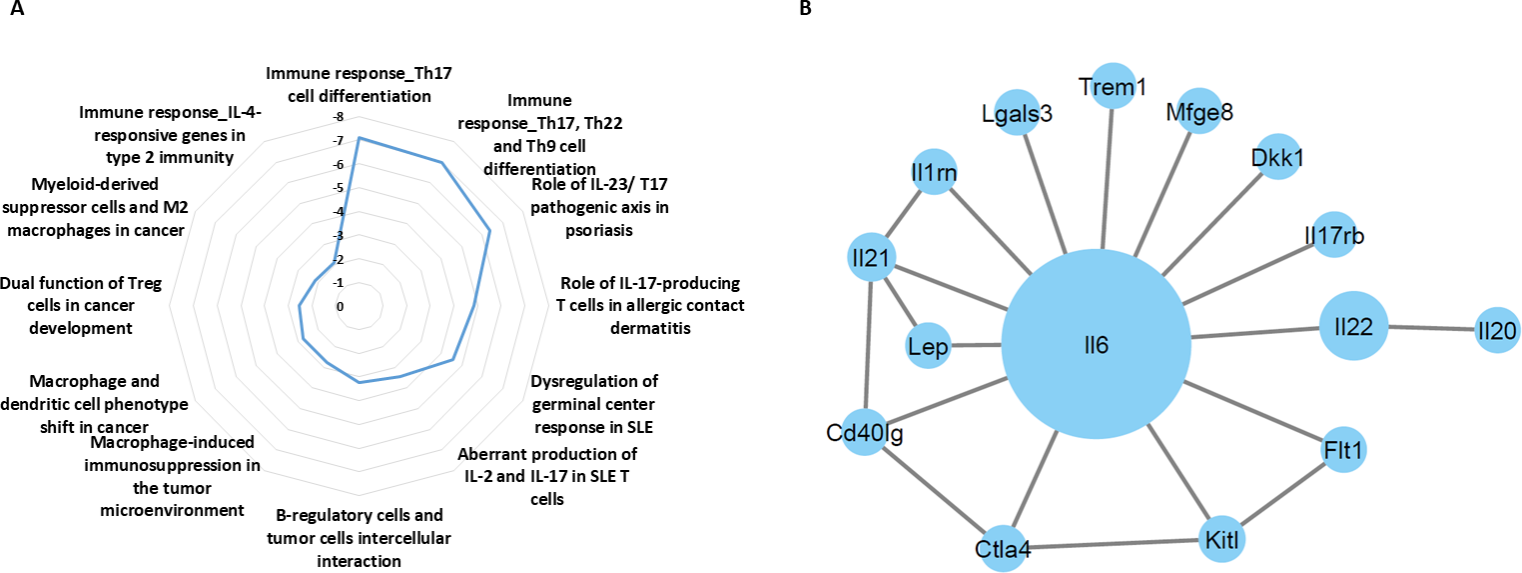
Anti-PD1 treatment induces changes in plasma proteomic profiles of mice. Tumor-free BALB/c mice were treated with anti-PD1 or IgG antibodies. One week later, plasma was extracted and the levels of 200 proteins were analyzed using antibody arrays. For each protein, plasma levels were expressed as fold change (FC) values (anti-PD1 vs IgG), shown in online supplemental table S4). (A) proteins exhibiting FC >1 were defined as differentially expressed proteins (DEPs). DEPs were analyzed to identify enriched biological pathways using the MetaCore pathway MAP tool and a radial plot. The significance values are calculated based on false discovery rate FDR adjusted p value. (B) The interactions between DEPs were mapped using Cytoscape. IL-17, interleukin; FDR, false discovery rate.

We next sought to identify the key proteins in the blood circulation driving treatment-induced aggressive properties of tumor cells. To this end, tumor-free BALB/c mice were treated with anti-PD1 or IgG control antibodies. One week later, plasma was extracted, pooled per group and analyzed using antibody arrays. The plasma levels of several proinflammatory associated cytokines including IL-6, IL-17, IL-21, IL-22 and interferon-γR1 were substantially increased in anti-PD1-treated mice in comparison to IgG-treated control mice (online supplemental table S4). These results suggest that anti-PD1 treatment promotes proinflammatory activity. The differentially expressed proteins were then analyzed using MetaCore bioinformatics software to identify enriched biological pathways. The analysis revealed multiple pathways associated with Th-17 immune response, as well as biological pathways associated with immunosuppressive effects mediated by macrophages and type 2 immunity (figure 4A). We next identified potential interactions between differentially expressed proteins exhibiting a fold change above 1. Cytoscape followed by centrality analysis revealed IL-6 as having the largest and most significant interaction network (figure 4B). The identified biological pathways and central proteins are known to support cancer progression.^34^ Thus, increased activity of these pathways and elevated levels of such factors can explain, at least partially, the host-mediated protumorigenic activities in response to anti-PD1 therapy.

### Blocking host-induced IL-6 in immunotherapy-treated mice improves therapeutic outcome

**Figure 5:**
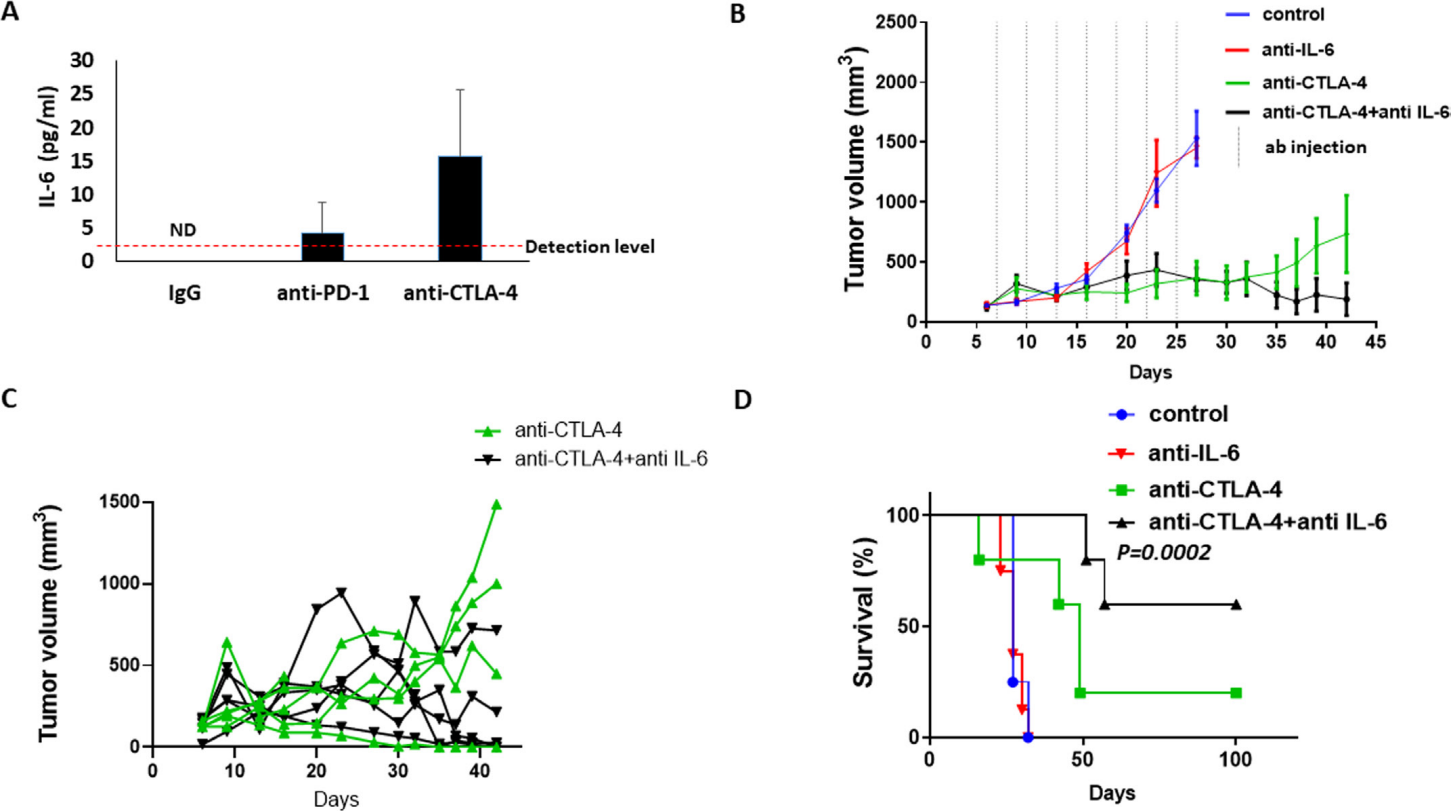
IL-6 blockade improves efficacy of anti-CTLA-4 treatment in mice bearing EMT6 tumors. (A) Tumor-free BALB/c mice were treated with IgG, anti-PD-1 or anti-CTLA-4 antibodies. One week later, plasma was collected. IL-6 plasma levels were quantified by specific ELISA. (B–D) EMT6 cells were implanted into the mammary fat pad of BALB/c mice (n=5–6 mice/group). When tumors reached ~100 mm^3^ (day 7) mice were treated with IgG, anti-CTLA-4, anti-IL-6 or a combination of anti-CTLA-4 and anti-IL-6 antibodies. Tumor growth was assessed over time (B). Spider plot showing tumor growth in individual mice treated with anti-CTLA-4 or the combination of anti-CTLA-4 and anti-IL-6 (C). A Kaplan-Meier survival curve is shown (D). IL-6, interleukin6; ND, non-detectable

Given that anti-PD1 treatment induces host-mediated tumor-supporting pathways, we hypothesized that inhibiting the key factors driving these pathways would improve the therapeutic efficacy of the ICI agent. The proteomic profiling analysis revealed that IL-6 was elevated in the plasma of anti-PD1-treated mice, and the bioinformatic analysis designated it as a key player in the Th17 protumorigenic pathway with the most significant interaction network (figure 4). We, therefore, investigated whether neutralizing IL-6 in combination with ICI therapy improves therapeutic outcome. First, using specific sensitive ELISA, we validated that IL-6 plasma levels are elevated in tumor-free mice treated with anti-PD1 and anti-CTLA-4 antibodies. In the IgG-treated control mice, IL-6 levels were undetectable, whereas in the anti-PD1-treated group levels of IL-6 were slightly above the detection level in some mice. Mice treated with anti-CTLA-4 antibodies exhibited a substantial increase in the plasma level of IL-6 compared with IgG control (figure 5A). We, therefore, opted to test the effect of inhibiting IL-6 in combination with anti-CTLA-4 treatment. To this end, mice were implanted with EMT6 cells, and when tumors reached a size of 50–100 mm^3^, treatment with anti-CTLA-4 or control IgG antibodies was initiated in the presence or absence of neutralizing anti-IL-6 antibodies. Tumor growth was assessed, and survival was monitored. In comparison to the IgG-treated control group, mice treated with anti-IL-6 alone did not show any therapeutic benefit, whereas mice treated with anti-CTLA-4 alone showed an initial benefit followed by an increased tumor growth rate from day 30. Interestingly, mice receiving the combination treatment of anti-CTLA-4 and anti-IL-6 antibodies exhibited sustained therapeutic benefit in comparison to mice treated with anti-CTLA-4 alone (figure 5B). Of note, as shown in the spider plot of tumor growth, only some mice displayed response to anti-CTLA-4 treatment, whereas the majority of mice displayed complete response to the combination treatment (figure 5C). In agreement with these results, mice treated with the combination of anti-CTLA-4 and anti-IL-6 antibodies exhibited the greatest survival benefit, reaching more than 100 days, whereas mice treated with anti-CTLA-4 monotherapy displayed a mild increase in survival in comparison to control IgG or anti-IL-6 monotherapy arms (figure 5D). In addition, in comparison to the in vivo findings, in vitro experiments testing the combination of IL-6 and anti-PD-1 further revealed that inhibiting IL-6 reverses anti-PD1-induced tumor cell aggressiveness to some extent. Specifically, plasma from combination-treated mice decreased invasive but not migratory properties of EMT6 cells in vitro, in comparison to plasma from mice treated with anti-PD1 monotherapy (online supplemental figure S5A). In addition, mice injected with EMT6 cells that were pre-cultured with plasma from combination-treated mice exhibited a decreased mortality rate in comparison to the anti-PD1 monotherapy group (online supplemental figure S5B). These collective results suggest that therapeutic outcome of ICI treatment can be improved by inhibiting therapy-induced, host-derived factors, in particular, those with key tumor-supporting roles.

### Protumorigenic activity is detected in NSCLC patient plasma early on during ICI therapy

**Figure 6:**
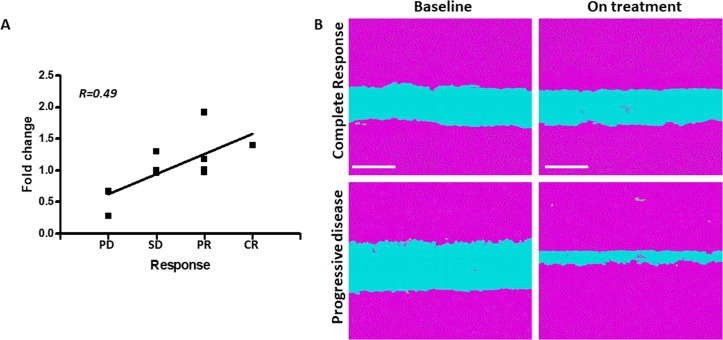
In vitro tumor cell motility in the presence of plasma from ICI-treated NSCLC patients correlates with therapy outcome. (A) Plasma samples were obtained from NSCLC patients undergoing anti-PD1- or anti-PD-L1-based immunotherapy (n=10) at baseline and on-treatment (2–3 weeks after the first treatment dose). The effects of the plasma samples on tumor cell motility were evaluated in a scratch wound assay with A549 NSCLC cell cultures. Gap closure was analyzed and expressed as a fold change value of average gap widths (on-treatment vs baseline) per patient. Correlation between gap closure and clinical response to treatment was plotted and evaluated. (B) Representative images at the 24-hour time point are shown for selected patients with complete response or progressive disease. Scale bar 300 µm. CR, complete response; ICI, immune checkpoint inhibitor; NSCLC, non-small cell lung cancer; PD, progressive disease; PR, partial response; SD, stable disease.

We next asked whether the tumor-supporting, host-mediated effects following ICI treatment can be detected in clinical samples. To this end, plasma samples were obtained from NSCLC patients undergoing anti-PD1- or anti-PD-L1-based immunotherapy (n=10). Patients’ age, treatment type and tumor grade are presented in online supplemental table S1. To differentiate between therapy-independent and therapy-induced effects, the plasma was sampled at two time points, namely, at baseline (before the commencement of treatment) and on-treatment (usually within 2–3 weeks after the first treatment dose). The effects of the plasma samples on tumor cell motility were evaluated in a scratch wound assay with A549 NSCLC cell cultures. Importantly, a moderate positive correlation was observed between worse clinical outcome and enhanced tumor cell motility induced by on-treatment plasma samples (figure 6, r=0.49). Of note, this experiment does not directly address the question of whether the host is responsible for inducing tumor aggressiveness in response to ICI therapy. Nevertheless, the results demonstrate that protumorigenic processes occur early on during ICI therapy, and that this occurrence correlates with worse clinical outcome in NSCLC patients. These findings are in line with our preclinical data, highlighting the possibility that ICI-induced tumor cell aggressiveness can be clinically relevant.

## Discussion

ICI-based immunotherapy has revolutionized clinical oncology in the last decade, yielding unprecedented durable remission in some patients with advanced metastatic disease. However, the proportion of ICI-treated patients deriving clinical benefit from this treatment modality is low across various cancer types, reaching 15% in melanoma and 20%–50% in NSCLC.^35 36^ In the last few years, a number of mechanisms have been suggested to explain resistance to ICI therapy. These include: tumor cell clones that have acquired immune-resistance^37^; ‘homeostatic resistance’ associated with the expression of immune checkpoints by tumor cells thus allowing them to escape the immune system^38^; and the recruitment of immunosuppressive cells to the tumor microenvironment which inhibit the activity of CTLs.^39 40^ Here, we describe an additional possible mechanism contributing to ICI therapy resistance. We demonstrate that host-derived factors are released into the circulation in response to ICI agents. These factors act directly on tumor cells, promoting migratory and invasive activities detected both in vitro (via Boyden chamber and scratch wound assays) and in vivo (via a pulmonary metastasis assay in mice). Thus, we have identified a therapy-induced, host-mediated effect that promotes tumor aggressiveness, potentially counteracting the therapeutic benefits of the ICI agent. Our previous studies have reported host-mediated, protumorigenic effects in response to chemotherapy, radiation, surgery and even targeted drugs.^22^ These protumorigenic host responses encompass a wide range of biological pathways, including angiogenesis, matrix metalloproteinase (MMP)-induced metastasis, M2-like macrophage activity and the secretion of specific cytokines and growth factors known to contribute to tumor growth.^41^ While our previous studies focused on treatment modalities that directly target the tumor, here we focus on agents that eradicate the tumor in an indirect manner, namely by enhancing antitumor immunity. The robust stimulation of the immune system by these agents is likely to generate excessive immune activities that not only increase the chance of autoimmunity,^42^ but also result in other unwanted effects, some of which promote tumor progression. Evidently, plasma from anti-PD1-treated immunocompromised SCID mice had no effect on tumor cell aggressiveness in contrast to plasma from anti-PD1-treated immunocompetent mice. In addition, CM of T cells obtained from spleens of anti-PD1-treated immunocompetent mice enhanced migratory and invasive properties of tumor cells, suggesting that T cells significantly contribute to the process. Indeed, previous studies have demonstrated that activated T cells secrete MMPs in order to infiltrate tumors through the basement membrane.^43^ It is, therefore, plausible that their activation may also promote prometastatic activity in tumors. Thus, the host response to ICI can be viewed as the ‘yin and yang’ effect, represented by the drug’s intended therapeutic effect and a reaction of the host that counteracts it, as described for various anticancer agents.^23^

The findings from our preclinical experiments raise the question of whether such effects occur in patients treated with ICI therapy. Recent clinical studies have demonstrated that some ICI-treated cancer patients exhibit rapid progression and acceleration of disease during treatment. This phenomenon is termed hyperprogressive disease (HPD). The percentage of such cases ranges between 4% and 29% depending on the cancer type, with head and neck cancer demonstrating the highest number of cases.^30 44^ These clinical scenarios necessitate the ability to identify predictive biomarkers for HPD, and its underlying mechanisms. Several mechanisms have been proposed to explain HPD. For example, in patients receiving ICI therapy as second-line treatment, discontinuation of the previous treatment may cause disease flare.^45^ In preclinical models, it has been shown that the combination of chemotherapy with ICI therapy contributes to the enrichment of resistant tumor clones that effectively escape the immune system, and therefore can explain HPD.^46^ Furthermore, a clinical study showed that specific tumor mutations in response to ICI therapy are associated with HPD.^47^ Our preclinical experiments described here show that the host response to ICI therapy promotes tumor aggressiveness, and therefore may potentially contribute to HPD. This possibility can be explored further by analyzing proteomic profiles of patient plasma samples before and during therapy, and identifying correlations with HPD.

Currently, there are major efforts underway to identify drug combinations for overcoming resistance to ICI therapy. Here, we propose a strategy for rationally designing such treatment combinations. In light of our preclinical findings showing that ICI treatment induces tumor-supporting biological pathways, we reason that inhibiting the key factors driving these pathways would potentially improve the therapeutic efficacy of the ICI agent. Our proteomic analysis shows that inflammatory associated biological pathways are activated in ICI-treated mice, with IL-6 serving as a potential hub or key factor that dominantly drives them. These results are in line with a number of studies demonstrating increased inflammation and autoimmune activities in patients receiving ICI therapy.^48^ Importantly, we show that therapeutic efficacy of anti-CTLA-4 is significantly improved by the coadministration of neutralizing antibodies against IL-6 in tumor-bearing mice. These results are in line with a previous publication demonstrating improved therapeutic outcome when anti-IL-6 is combined with anti-PD1 or anti-PD-L1 treatment.^49^ Moreover, our in vitro experiments demonstrate that inhibiting IL-6 diminishes anti-PD-1-induced tumor cell invasive properties, further supporting the notion that blocking specific therapy-induced host factors represents a strategy for overcoming therapy resistance. It should be noted that in the clinic, the blockade of IL-6 in combination with ICI therapy is currently under investigation (NCT03999749, ClinicalTrials.gov).

In summary, our preclinical study reveals a systemic host-mediated response to ICI agents that promotes tumor cell aggressiveness, and potentially counteracts the therapeutic benefit of the drug. Our findings have clinical ramifications, both for the discovery of novel biomarkers for predicting clinical response to ICI therapy, as well as for the rational design of combination therapies with improved outcomes.
