# Supplementary material for: Host response to immune checkpoint inhibitors contributes to tumor aggressiveness
Source: J Immunother Cancer. 2021 Mar 11;9(3):e001996. doi: 10.1136/jitc-2020-001996 (PMC7957134; doi:10.1136/jitc-2020-001996)
Supplement: Supplementary data [file jitc-2020-001996supp001.pdf]

Supplemental Information Online

Irena Khononov et al.,

Supplemental Materials and Methods

Migration and invasion assay

Serum-starved EMT6, RET, B16 or LLC cells ($2x10^5$ cells in 0.2 ml serum free DMEM medium) were added to the upper compartment of a Boyden chamber that was coated with either 50µl Matrigel (BD Biosciences, Franklin Lakes, NJ) for invasion assays or 100µl fibronectin (10 µg/ml) for migration assays. The lower compartment was filled with serum-free DMEM medium containing 5% plasma drawn from mice treated with IgG, anti-PD1, anti-CTLA-4, anti-IL-6, or a combination of anti-IL-6 and anti-PD-1 antibodies, or with 100% conditioned medium of isolated T cells from the spleens of IgG- or anti-PD1-treated mice, as previously described [1]. In some experiments, anti-PD1 or IgG antibodies (0.5µg/ml) were included in the upper chamber to test direct effect of the antibody on tumor cells or in the lower chamber to test the agent's chemotaxis properties. After 16 hours, cells that migrated to the bottom filter were stained with crystal violet and counted under an inverted microscope (Leica DMIL LED, Leica mycrosystems, Wetzler, Germany) per x200 objective-field. At least 5 fields per assay were analyzed. Three biological repeats of each experiment were performed.

Scratch wound assay

The effect of plasma from mice or patients on tumor cell motility was evaluated by a scratch wound assay, as previously described [2]. Briefly, a 'scratch' was made on confluent cultures of EMT6, B16 or RET cells grown in a 96-well plate using the IncuCyte Zoom HD/2CLR system (Essen BioScience, Ann Arbor, MI). The cells were then incubated with medium supplemented with 5% plasma from IgG- or anti-PD1-treated mice. A549 cells were used according to the same protocol in the presence of 5% baseline or on-treatment plasma samples from ICI-treated NSCLC patients. Cell migration into the 'scratch' was monitored, and analyzed by IncuCyte Zoom 2016B software. For mice plasma, percentages of wound confluence were plotted. For patient plasma, the average width of the gap was quantified. Fold change values of average gap widths (on-treatment vs baseline) were calculated per patient. Correlation between gap closure and clinical response to treatment was evaluated. All experiments were performed in triplicate.

Flow cytometry analysis

Matrigel plugs, EMT6 tumors, and peripheral blood were collected from mice and samples were prepared as single cell suspensions as previously described [3]. The single cell samples were immunostained with various antibodies to identify different immune cell types, as indicated in Table S2. All antibodies were monoclonal, purchased from BD Biosciences, and BioLegend (San Diego, CA), and used in accordance with the manufacturer's instruction. At least 150,000 events were acquired using a Fortessa flow cytometer and analyzed by FlowJo 7.6.1. Software.

Mass cytometry

EMT6 tumors and peripheral blood from IgG- and anti-PD1-treated mice were harvested two weeks after treatment was initiated. Single cell suspensions were prepared as previously described [3]. Single cell samples were labelled with a unique binary combination of mass-tags before combining the cells into a single pool for antibody staining. Metal labelled antibodies as indicated in Table S3 were added to the samples. All antibodies were conjugated using the MAXPAR reagent (Fluidigm, South San Francisco, CA) and titered prior to staining. Rhodium and iridium intercalators were used to identify live/dead cells. Cells were then washed twice with PBS, fixed in 1.6% formaldehyde (SigmaAldrich, St. Louis, MO), washed again in ultrapure H_2O, and acquired by CyTOF mass cytometry system (Fluidigm). Acquired data was uploaded to Cytobank web server (Cytobank Inc.). Data analysis was performed by viSNE algorithm [4], via the Cytobank server. Changes in specific populations were validated by flow cytometry, as described above.

Cell viability by AlamarBlue assay

Cell viability was assessed using the metabolic indicator dye AlamarBlue (AbD Serotech Ltd., Oxon Kidlington.UK) as described [5]. Briefly, EMT6 or RET cells were cultured in a 96 well plate, in the presence of conditioned medium of T cells isolated from spleens of anti-PD1- or IgG-treated mice as previously described [1]. Ten percent AlamarBlue reagent was added to each well. Absorbance was measured using a fluorescence plate reader (TECAN M200, Mannedorf, Switzerland) with wavelengths 570nm and 600nm as references. Results are presented as the percentage of AlamarBlue reduction during the first 72-hour time period and corrected to the absorbance values of negative controls. All experiments were carried out in triplicate.

Protein array and ELISA

Tumor-free BALB/c mice were treated with IgG or anti-PD1 antibodies for one week as described in the Methods section of the main text. Plasma was obtained, pooled per group (n=5 mice/group),

and applied to Proteome Profiler Mouse XL Cytokine Array chips (Cat. ARY028, RayBiotech Life, Norcross, GA) in accordance with the manufacturer's instruction. The signals corresponding to each factor in the array were quantified by Raybio® Q-Analyzer. Fold changes in the level of each protein were determined by calculating the ratio between anti-PD1:IgG values. Biological pathways associated with the differentially expressed proteins were identified using MetaCore pathway map tool (Clarivate analytics, London, UK). Proteins with fold change (anti-PD1 to IgG ratio) above 1.0 were further examined for protein-protein interactions using STRING database [6]. Protein network was generated using Cytoscape [7]. The size of the nodes is based on ‘betweenness’ centrality measure. The plasma levels of IL-6 in IgG- anti-PD1-and anti-CTLA-4-treated mice were evaluated using a mouse IL-6 specific enzyme-linked immunosorbent assay (ELISA) kit (Cat AB-ab100712, Abcam, Cambridge, UK). The ELISA experiments were carried out in triplicate, and analyzed as mean ± SD.

References

1. Kan T, Feldman E, Timaner M, Raviv Z, Shen-Orr S, Aronheim A, Shaked Y (2020) IL-31 induces antitumor immunity in breast carcinoma. J Immunother Cancer 8 (2).
2. Margiotta A, Progida C, Bakke O, Bucci C (2017) Characterization of the role of RILP in cell migration. Eur J Histochem 61 (2):2783.
3. Timaner M, Beyar-Katz O, Shaked Y (2016) Analysis of the Stromal Cellular Components of the Solid Tumor Microenvironment Using Flow Cytometry. Curr Protoc Cell Biol 70:19 18 11-19 18 12.
4. Amir el AD, Davis KL, Tadmor MD, Simonds EF, Levine JH, Bendall SC, Shenfeld DK, Krishnaswamy S, Nolan GP, Pe'er D (2013) viSNE enables visualization of high dimensional single-cell data and reveals phenotypic heterogeneity of leukemia. Nat Biotechnol 31 (6):545-552.
5. Gingis-Velitski S, Loven D, Benayoun L, Munster M, Bril R, Voloshin T, Alishekevitz D, Bertolini F, Shaked Y (2011) Host response to short-term, single-agent chemotherapy induces matrix metalloproteinase-9 expression and accelerates metastasis in mice. Cancer Res 71 (22):6986-6996.
6. von Mering C, Jensen LJ, Snel B, Hooper SD, Krupp M, Foglierini M, Jouffre N, Huynen MA, Bork P (2005) STRING: known and predicted protein-protein associations, integrated and transferred across organisms. Nucleic Acids Res 33 (Database issue):D433-437.
7. Shannon P, Markiel A, Ozier O, Baliga NS, Wang JT, Ramage D, Amin N, Schwikowski B, Ideker T (2003) Cytoscape: a software environment for integrated models of biomolecular interaction networks. Genome Res 13 (11):2498-2504.

Supplemental Tables and Figures

Table S1: NSCLC patient characteristics

Age	Sex	Grade	Therapy	Response
70	Male	IV	Pembrolizumab	Stable disease
53	Female	IV	Pembrolizumab	Partial response
67	Male	IIIB	Avelumab	Stable disease
60	Male	IV	Durvalumab + Gemcitabine + Cisplatine followed by Durvalumab	Partial response
68	Male	IV	Durvalumab + Carboplatine + Pemetrexed followed by Durvalumab + Pemetrexed	Stable disease
61	Male	IV	Tremelimumab + Durvalumab + Pemetrexed + Carboplatine followed Tremelimumab + Durvalumab followed by Durvalumab	Partial response
66	Female	IV	Pembrolizumab	Complete response
69	Female	IV	Pembrolizumab	Progressive disease
63	Female	IV	Pembrolizumab	Progressive disease
71	Female	IV	Pembrolizumab	Partial response

Table S2: Immune cell surface marker definition

**Immune cell**	**Surface markers**
Th cells	$CD45^{+}CD4^{+}$
Activated Th cell	$CD45^{+}CD4^{+}CD25^{+}$
Treg	$CD45^{+}CD4^{+}CD5^{+}$
CTL	$CD45^{+}CD8^{+}$
Activated CTL	$CD45^{+}CD8^{+}CD25^{+}$
Natural Killer cells	$CD45^{+}NKp46^{+}$
B cells	$CD45^{+}CD19^{+}$
Dendritic cells	$CD45^{+}CD11c^{+}$ $F4/80^{-}$ $GR1^{-}$
M1 macrophages	$CD45^{+}CD11b^{+}F4/80^{+}CD11c^{+}CD206^{-}$
M2 macrophages	$CD45^{+}CD11b^{+}F4/80^{+}CD11c^{-}CD206^{+}$
Monocytic MDSC	$CD45^{+}CD11b^{+}F4/80^{-}GR1^{+}Ly6C^{high}$
Granulocytic MDSC	$CD45^{+}CD11b^{+}F4/80^{-}GR1^{+}Ly6C^{Low}$

Table S3: Antibody panel used for CyTOF

#	Metal	Ab1	Clone	Supplier	Catalog no.
**1**	115In	CD45	30-F11	Biolegend	103120
**2**	141Pr	CD80	16-10A1	Biolegend	104702
**3**	142Nd	GR1	RB6-8C5	Biolegend	108402
**4**	143Nd	IL-10r	1B1.3a	Biolegend	112708
**5**	144Nd	F4/80	BM8	Biolegend	123102
**6**	145Nd	CD4	RM4-5	Biolegend	100520
**7**	146Nd	CD45R	RA3-6B2	Biolegend	103202
**8**	147Sm	Ly6c	HK1.4	Biolegend	128002
**9**	148Nd	IL-6r (CD126)	D7715A7	Biolegend	115808
**10**	149Sm	CD8	53-6.7	Biolegend	100716
**11**	150Nd	Nkp46	29A1.4	Biolegend	137625
**12**	151Eu	CD206	C068C2	Biolegend	141702
**13**	152Sm	CD25	3C7	Biolegend	101913
**14**	153Eu	CD14	Sa14-2	Biolegend	123302
**15**	154Sm	CD11c	N418	Biolegend	117302
**16**	155Gd	CCR9	9B1	Biolegend	129704
**17**	156Gd	CD49b	HMα2	Biolegend	103513
**18**	157Gd	CD19	6D5	Biolegend	115502
**19**	158Gd	CD279 (PD1)	RMP1-14	Biolegend	114102
**20**	159Tb	CD27	LG.3A10	Biolegend	120101
**21**	160Gd	CD69	H1.2F3	Biolegend	104502
**22**	161Dy	CD150	TC15-12F12.2	Biolegend	115933
**23**	162Dy	CD31	MEC 13.3	BD Biosciences	553370
**24**	163Dy	CD127	A7R34	Biolegend	135002
**25**	164Dy	CD28	37.51	Biolegend	102102
**26**	165Ho	CD115	AFS98	Biolegend	135502
**27**	166Er	SiglecF	238023	R&D Systems	MAB1706
**28**	167Er	CD93	223437	R&D systems	MAV1696
**29**	168Er	CD117	2B8	Biolegend	105802
**30**	169Tm	Tim1	RMT1-4	Biolegend	119502
**31**	170Er	CD62L	MEL-14	Biolegend	104402

**32**	171Yb	CD44	IM7	Biolegend	103014
**33**	172Yb	CD23	B3B4	Biolegend	101625
**34**	173Yb	Sca-1	D7	Biolegend	108102
**35**	174Yb	Vegfr2	HP6017	Biolegend	409302
**36**	175Lu	CD5	53-7.3	Biolegend	100602
**37**	176Yb	CD11b	M1/70	Biolegend	101202

Table S4: Fold change in the level of circulating proteins in mice (anti-PD1 vs IgG treatment)

Protein	FC
I-TAC	9.26
IL-22	9.12
IL-1ra	8.72
IFNg R1	5.83
ADAMTS1	4.84
TREM-1	3.90
Prostasin	3.86
AR	3.74
CD40L	3.63
IL-3 Rb	3.44
Fas	3.43
Artemin	3.42
IL-17B	3.41
ALK-1	3.38
MFG-E8	2.75
Persephin	2.72
VEGF R1	2.64
IL-21	2.63
CTLA4	2.42
Lymphotactin	2.36
Epigen	2.28
Galectin-3	2.28
IL-6	2.14
TPO	2.14
Leptin	2.08
IL-17B R	2.06
IL-20	2.04
Dkk-1	2.03
SCF	2.01
ANGPTL3	1.95
Gremlin	1.85
Fractalkine	1.81
IL-1 R4	1.78
IL-2 Ra	1.73
HGF R	1.70
TROY	1.67
BAFF R	1.61
TCA-3	1.60
IL-17F	1.57
IL-17	1.56
HAI-1	1.54
MCSF	1.52
IL-23	1.50
TARC	1.50
IL-12p70	1.47
VEGF	1.46
TRANCE	1.45
LIX	1.42
MIP-3a	1.41
BLC	1.41
Shh-N	1.40

TACI	1.39
MMP-2	1.39
IL-12p40	1.38
IL-1b	1.38
IGFBP-5	1.38
TNFa	1.35
CD27	1.33
MMP-10	1.33
MCP-1	1.33
TWEAK R	1.33
IGFBP-2	1.32
TGFb1	1.30
PF4	1.30
Adiponectin	1.29
Granzyme B	1.29
TWEAK	1.24
IL-3	1.24
Osteoactivin	1.23
Chordin	1.23
Clusterin	1.20
Pentraxin 3	1.20
Leptin R	1.18
ICAM-1	1.15
L-Selectin	1.13
IGFBP-6	1.13
HGF	1.13
OPN	1.12
IFNg	1.12

SDF-1a	1.12
TECK	1.11
CRP	1.10
MadCAM-1	1.10
IL-10	1.09
Flt-3L	1.09
JAM-A	1.08
MIP-1g	1.08
TremL1	1.08
E-Cadherin	1.08
IL-7	1.08
GM-CSF	1.07
VCAM-1	1.06
VEGF-D	1.06
IL-5	1.05
G-CSF	1.05
Galectin-1	1.04
MIG	1.04
MIP-1a	1.00
CCL28	0.99
Decorin	0.98
Cystatin C	0.98
MIP-3b	0.97
KC	0.97
Fas L	0.97
MMP-3	0.96
IL-15	0.96
SLAM	0.96

CXCL16	0.96
Periostin	0.95
ACE	0.95
E-selectin	0.95
Nope	0.95
IL-1a	0.94
Gas 1	0.94
VEGF R2	0.93
PDGF-AA	0.93
IGFBP-3	0.92
MIP-1b	0.91
CD36	0.89
TCK-1	0.88
MDC	0.88
CCL6	0.88
IL-4	0.88
Resistin	0.87
Epiregulin	0.87
Eotaxin	0.86
VEGF-B	0.86
CT-1	0.86
MBL-2	0.85
IL-28	0.85
Eotaxin-2	0.85
TNF RII	0.84
P-Cadherin	0.84
P-selectin	0.84
Chemerin	0.82

IL-13	0.82
Gas 6	0.82
Axl	0.82
IL-2	0.81
TNF RI	0.81
CD40	0.80
OPG	0.80
IL-17E	0.79
CD30	0.79
Dtk	0.79
MCP-5	0.79
Lipocalin-2	0.78
EGF	0.78
Neprilysin	0.78
GITR L	0.77
Endoglin	0.76
Meteorin	0.75
sFRP-3	0.73
gp130	0.73
Renin 1	0.64
Prolactin	0.64
4-1BB	0.61
CD48	0.60
RAGE	0.56
IL-33	0.56
Pro-MMP-9	0.54
CD6	0.51
RANTES	0.51

Fcg RIIB	0.49		C5a	0.30
Progranulin	0.42		bFGF	0.27
Galectin-7	0.39		IGF-1	0.25
Fetuin A	0.36		VEGF R3	0.06

Tumor-free BALB/c mice were treated with anti-PD1 or IgG antibodies. One week later, plasma was extracted and pooled per group. The levels of the indicated proteins were analyzed using antibody arrays. Fold change (FC) values (anti-PD1 to IgG) were calculated per protein.

Supplemental Figures

Figure S1: Assessment of tumor cell migration and invasion in vitro under various conditions. (A) Mice bearing RET or B16 melanoma tumors were treated with anti-CTLA-4 or control IgG antibodies. One week later, mice were sacrificed, and plasma was collected. Migratory (left) and invasive (right) properties of RET and B16 cells were assessed in Boyden chamber assays in the presence of plasma extracted from IgG- or anti-CTLA-4-treated mice. Representative images are shown. Scale bar, 200 µm. Cell coverage was quantified from the images (n=5-8 fields/ group). (B) EMT6 cells were placed in the upper compartment of a Boyden chamber, and anti-PD1 or IgG control antibodies (0.5µg/ml) were either placed in the upper compartment (to assess possible direct effects of the antibodies) or in the lower compartment (to assess chemotaxis). Migration and invasion of tumor cells were evaluated. Representative images are shown. Scale bar 200 µm. Cell coverage was quantified from the images (n=5-8 fields/ group). (C) Tumor-free mice were treated with anti-PD1 or control IgG antibodies. One week later, mice were sacrificed, and plasma was collected. Migratory (left) and invasive (right) properties of EMT6 cells were assessed in Boyden chamber assays in the presence of plasma extracted from IgG- or anti-PD1-treated mice. Representative images are shown. Scale bar, 200 µm. Cell coverage was quantified from the images (n=5-8 fields/ group). Statistical significance was assessed by unpaired two-tailed t-test. Significant p values are shown as * p<0.05; ** p<0.01; *** p<0.001.

Figure S2: Pulmonary metastasis assay in mice pre-treated with anti-PD1 or IgG antibodies. Tumor-free BALB/c and C57BL/6 mice were treated with anti-PD1 or IgG antibodies for one week. Subsequently, EMT6, B16, RET or LLC cells (50,000 cells/mouse) were injected through the tail vein to generate a pulmonary metastasis model. Kaplan Meier survival curves are shown.

Figure S3: Immune cell composition in Matrigel plugs containing plasma from anti-PD1 treated mice. RET tumor-bearing C57BL/6 mice were treated with IgG control or anti-PD1 antibodies. One week later, mice were sacrificed, and plasma was collected. The plasma was mixed with Matrigel in a 1:10 ratio and the mixture was implanted into the flanks of naïve C57BL/6 mice. After 10 days, Matrigel plugs were removed. (A) Sections of Matrigel plugs were stained with H&E. Representative images are shown. Scale bar 200 µm. (B-C) In a parallel experiment, Matrigel plugs were prepared as single cell suspensions and evaluated by flow cytometry. Absolute numbers of lymphoid (B) and myeloid (C) immune cells per mg Matrigel are presented. Statistical

significance was assessed by unpaired two-tailed t-test. Significant p values are shown as * p<0.05; ** p<0.01; *** p<0.001.

Figure S4: Flow cytometry validation of immune cell composition in the EMT6 tumors and blood of anti-PD1 and IgG treated mice. EMT6 tumor-bearing BALB/c mice were treated with IgG control or anti-PD1 antibodies (n=5 mice/group). One week later, blood was drawn, and tumors were removed and prepared as single cell suspensions. Samples were acquired by flow cytometry using the combination of antibodies indicated in Table S2. The percentages of each immune cell type out of total immune cells are shown. Statistical significance was assessed by unpaired two-tailed t-test. Significant p values are shown as * p<0.05; ** p<0.01; *** p<0.001.

Figure S5: IL-6 inhibition counteracts anti-PD-1-induced metastatic properties of tumor cells. Tumor-free BALB/c mice were treated with anti-IL-6, anti-PD-1 or a combination of anti-IL-6 and anti-PD-1 antibodies. Control mice were treated with IgG antibodies. One week later, mice were sacrificed, and plasma was collected. (A) Migratory and invasive properties of EMT6 cells were assessed in Boyden chamber assays in the presence of plasma extracted from the treated mice. Representative images are shown. Scale bar, 200 µm. Cell coverage was quantified from the images (n=4-5 fields/ group). (B) EMT6 cells were cultured for 4 hours in the presence of plasma extracted from the treated mice. The cells were then injected through the tail vein to naïve mice (n=7-8 mice/group), and survival was monitored. Kaplan-Meier curves are shown. Statistical significance was assessed by one way ANOVA followed by Tukey post-test. Significant p values are shown as *** p<0.001 from control or otherwise indicated in the figure.

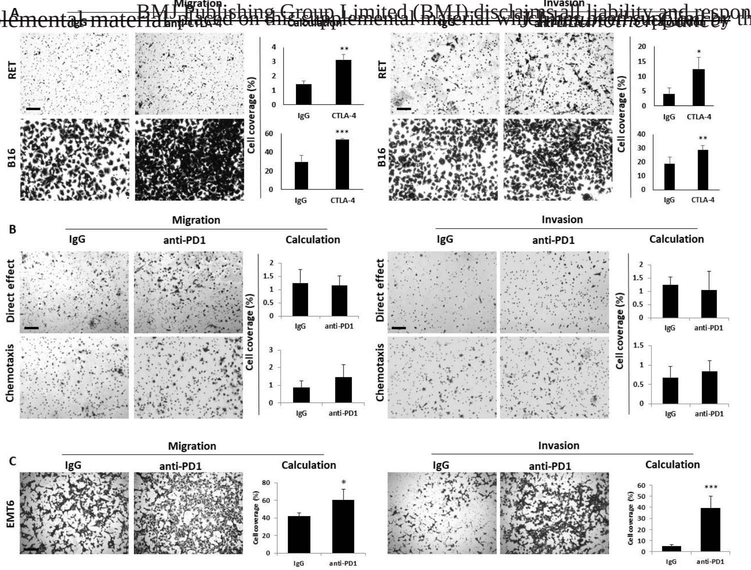
A
Migration
IgG
anti-CTLA-4
Calculation
Invasion
RET
B16
Cell coverage (%)
IgG
CTLA-4
B
Migration
Invasion
IgG
anti-PD1
Calculation
Direct effect
Chemotaxis
C
EMT6

Figure S2

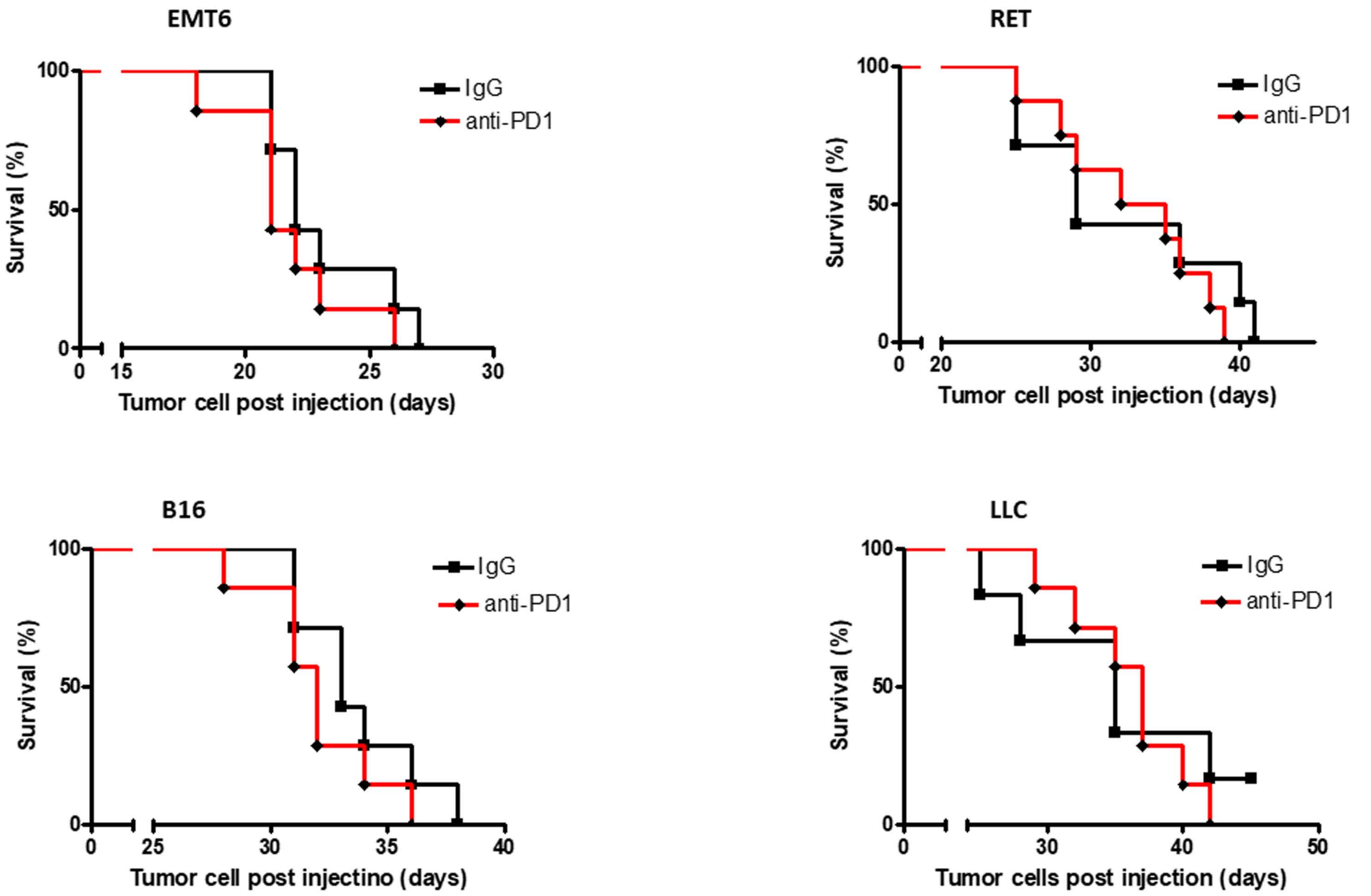
EMT6
IgG
anti-PD1
Survival (%)
100
50
0
0
15
20
25
30
Tumor cell post injection (days)
RET
IgG
anti-PD1
Survival (%)
100
50
0
0
20
30
40
Tumor cell post injection (days)
B16
IgG
anti-PD1
Survival (%)
100
50
0
0
25
30
35
40
Tumor cell post injectino (days)
LLC
IgG
anti-PD1
Survival (%)
100
50
0
0
30
40
50
Tumor cells post injection (days)

Figure S3

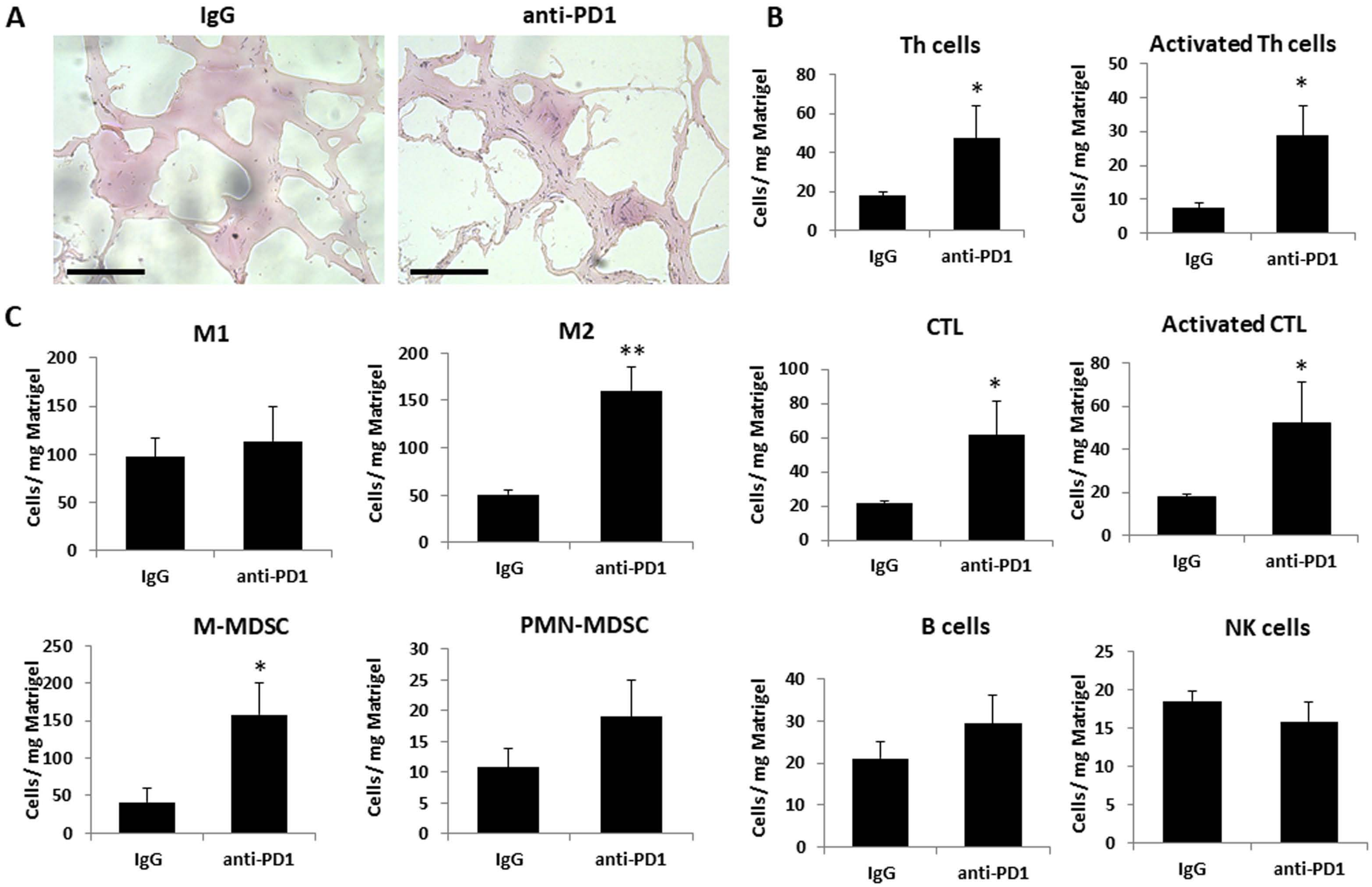
A
IgG
anti-PD1
B
Th cells
Activated Th cells
C
M1
M2
CTL
Activated CTL
M-MDSC
PMN-MDSC
B cells
NK cells
Cells / mg Matrigel
IgG
anti-PD1
*
**

BMJ Publishing Group Limited (BMJ) disclaims all liability and responsibility arising from any reliance placed on this supplemental material which has been supplied by the author(s)

Figure S4

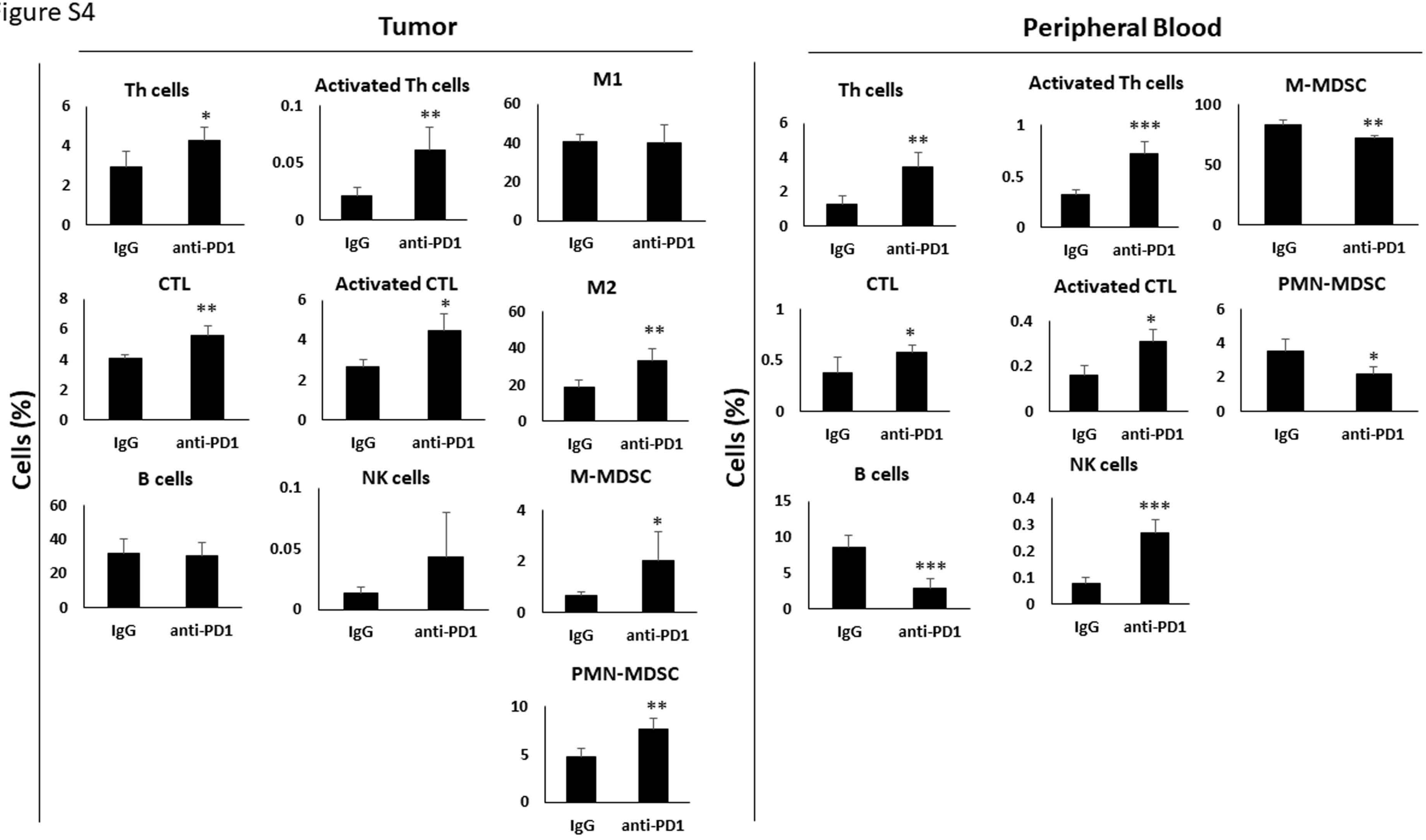

Figure S5

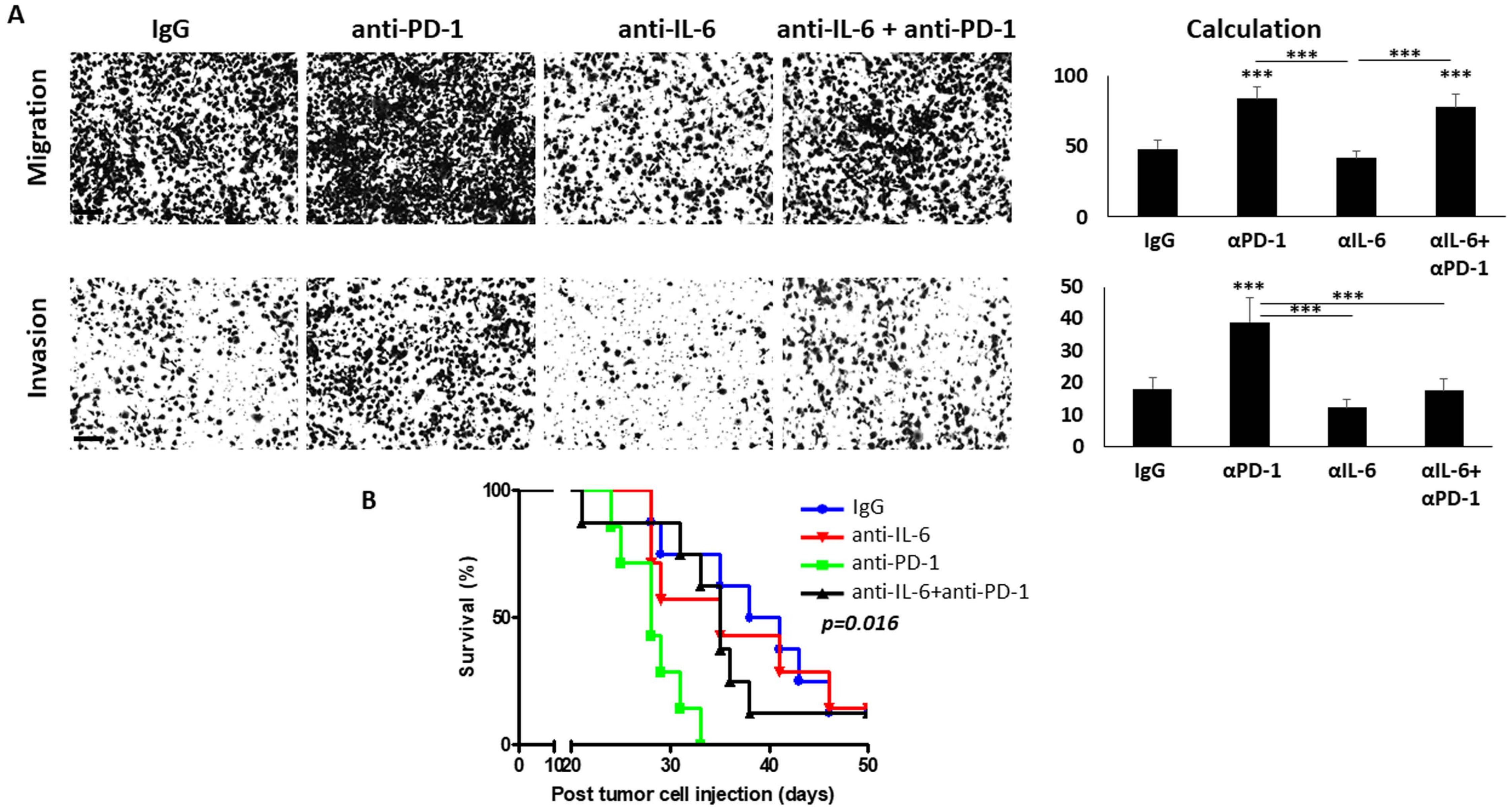
A
IgG
anti-PD-1
anti-IL-6
anti-IL-6 + anti-PD-1
Calculation
Migration
Invasion
***
100
50
0
IgG
αPD-1
αIL-6
αIL-6+
αPD-1
50
40
30
20
10
0
B
Survival (%)
Post tumor cell injection (days)
0
10
20
30
40
50
IgG
anti-IL-6
anti-PD-1
anti-IL-6+anti-PD-1
p=0.016
